# Perspectives of community pharmacy staff on commonly encountered skin conditions and the key challenges towards enhancing their role in dermatology

**DOI:** 10.1002/ski2.369

**Published:** 2024-03-11

**Authors:** Zakia Shariff, Jane Harvey, Paul Leighton, Matthew Boyd, Matthew Ridd, Miriam Santer, Rod Tucker, Ian Maidment

**Affiliations:** ^1^ Aston Pharmacy School Aston University Birmingham UK; ^2^ Centre for Evidence Based Dermatology University of Nottingham Nottingham UK; ^3^ School of Pharmacy University of Nottingham Nottingham UK; ^4^ Population Health Sciences, Bristol Medical School University of Bristol Bristol UK; ^5^ Primary Care Research Centre University of Southampton Southampton UK; ^6^ School of Life Sciences University of Bradford Bradford UK

## Abstract

This research letter discusses the perspectives of community pharmacy staff on commonly encountered skin conditions and the key challenges towards enhancing their role in this area. A mixed methods online survey was created, and a total of 174 community pharmacy staff completed the survey. The results highlight the range of conditions currently encountered in community pharmacy and the breadth of challenges facing community pharmacy staff, in particular challenges surrounding providing a differential diagnosis. Community pharmacies are an integral part of the NHS and have a key role in managing skin conditions; however, in order to optimise this role, the perspectives of staff discussed in this letter need to be further explored and addressed.

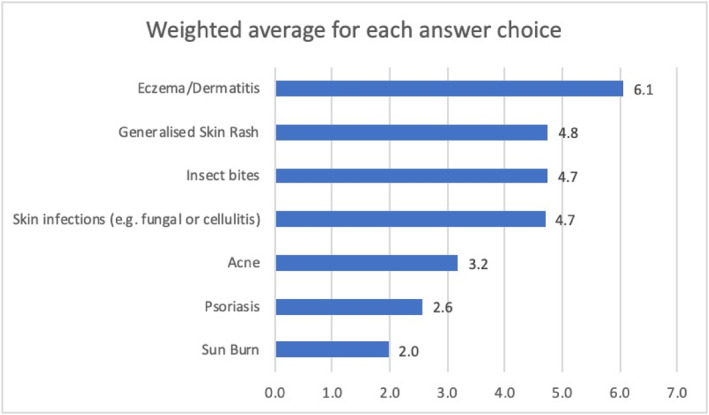

Dear Editor,

Skin disease is amongst the most prevalent of diseases in the UK; at any one time 54% of the population are affected costing the NHS in England and Wales £1820 million annually.^1^ Most people (69%) with skin conditions rely on self‐care.^2^ Community pharmacy provides an accessible and convenient place for patients to initially access appropriate support.^3^ Due to the wide range of skin diagnoses, community pharmacists' UK role involves diagnosing and giving advice on the management of common skin conditions,^4^, ^5^ providing advice on the use of over the counter (OTC) products, overseeing the management of skin conditions by prescribed treatments (e.g., providing counselling at the point of dispensing) and providing onward referrals when necessary. While this is a multidimensional role, there is limited research in this area, especially from the perspectives of community pharmacy staff.

Considering the need for further research, this exercise aimed to explore the perspectives of community pharmacy staff on commonly encountered skin conditions and the key challenges towards enhancing their role in this area. A steering group containing experts in community pharmacy, primary care dermatology and priority setting exercises supported this exercise and was formed by professional links primarily via the Society for Academic Primary Care Dermatology Special Interest Group. An online survey, using the JISC Online Survey platform, was created. After initial piloting, the online survey was distributed by Twitter®, general snowballing techniques (for example re‐tweeting), via professional networks and via the Pharmaceutical Services Negotiating Committee newsletter.

In total 174 community pharmacy staff (67.8% female, 29.3% male, 2.9% prefer not to say) completed the survey (169 over a period of 6 months [20.7.2021–20.1.2022] plus five involved in the pilot). Participants from a range of ethnic backgrounds participated in the study including White British (34.5%, *n* = 60), Asian British‐ Indian (21.8%, *n* = 38) and Asian British‐ Pakistani (10.3%, *n* = 18).

First, participants were asked to rank seven skin conditions commonly encountered in community pharmacy, with one being the most frequently encountered and seven being the least. A weight was assigned to each condition; the condition that most participants ranked as 1 was assigned a weight of 7 while that which most participants ranked as 7 was assigned a weight of 1. The average weight for each condition was then calculated as follows:

$$\frac{x_{1}w_{1}+x_{2}w_{2}+x_{3}w_{3}+x_{4}w_{4}+x_{5}w_{5}+x_{6}w_{6}+x_{7}w_{7}}{174}$$

where *w* = weight of ranked position; *X* = response count for answer choice.

The weighted average as calculated for each condition can be found in Figure 1.

**FIGURE 1:**
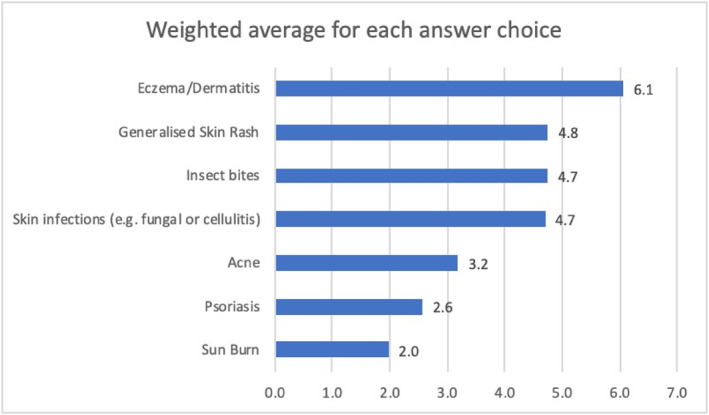
Weighted average for each answer choice (*n* = 174).

Next, participants listed any other skin conditions commonly encountered. A total of 65 responses were provided, highlighting the large range of skin conditions currently encountered. Responses included: infectious diseases affecting the skin (e.g., shingles, impetigo, chicken pox, hand foot and mouth, cold sores, athletes foot); reactions to external stimuli (e.g., burns, UV damage, solar keratosis); dermatitis/eczema related conditions (e.g., seborrhoeic dermatitis); urticarias, erythemas and other inflammatory dermatoses (e.g., hives, rosacea, urticaria); disorders of skin colour (e.g., vitiligo, hyperpigmentation); disorders of skin appendages (e.g., head and body hair loss); allergic reactions; skin lesions; skin cancer; boils; warts; verruca; corn; callouses and moles.

Finally, respondents listed up to two of the most challenging aspects of treating or managing patients presenting with skin conditions in the challenges section. Content analysis was used to analyse the data, providing a list of five most commonly reported challenges:

(1) Ability to provide a differential diagnosis (69).
(2) Knowledge of when and how to refer skin conditions (53).
(3) Difficulties related to the availability of OTC treatment options (41).
(4) Ability to choose and provide patients with the most appropriate treatment option (40).
(5) Challenges in relation to the patient consultation, including challenges communicating when using photos/telephone consultations, and challenges arising due to the sensitivity surrounding the topic of skin conditions (24).

These perspectives highlight the breadth of challenges facing community pharmacy staff. The large variation in symptoms and severity of skin disorders leads to difficulties in the first instance in providing a differential diagnosis. Generalised skin rash in particular, ranked as the second most commonly encountered condition in the present study, can have a range of common differentials, such as contact dermatitis, atopic eczema, or manifestation of systemic disease.^6^

There is particularly a need to focus on improving the ability to diagnose minor ailments commonly encountered in community pharmacy. Eczema/dermatitis was ranked as the most commonly encountered skin condition, and these results are supported by previous studies exploring pharmacists' perceptions on the range of skin conditions they encounter.^5^ Improving diagnostic ability for commonly encountered conditions can help enhance the role of community pharmacy in dermatology; however, there is then a need to choose an appropriate available treatment option—a further challenge identified in the present study.

Pharmacists also have a key role to play in providing counselling and advice to patients; adherence is a growing area of concern in the treatment of skin diseases.^7^ However time constraints and a limited knowledge of dermatology may be barriers towards implementing this role.^8^ The present study has highlighted some of the further challenges experienced by community pharmacy staff in relation to the patient consultation; participants highlighted difficulties with patients not adhering to or understanding treatment regimes, difficulties diagnosing skin conditions from photos/over the telephone and difficulties providing consultations due to the sensitive nature of some skin conditions.

We acknowledge that this study has some limitations, including the need for a broader survey sample, the potential for responder bias due to the recruitment strategy and a more vigorous methodological approach to explore this topic further. This survey, however, is an important conversation starter to illustrate the complexities surrounding the role of community pharmacy in skincare. A project was subsequently commissioned by the NIHR School for Primary Care Research which builds on the results of this survey and explores further some of the potential areas for research to support community pharmacies in managing skin conditions.^9^

In conclusion, this survey has highlighted the key challenges facing community pharmacy staff in the area of dermatology. The present study highlights the need for greater support for community pharmacy staff in (1) the provision of a differential diagnosis (2) guidance on when to refer, including training resources that are inclusive of skin of colour, (3) review of availability of OTC treatments for common skin conditions so that pharmacists can provide help when appropriate, and (4) resources for pharmacists to share with patients to support messages around treatments and treatment adherence. Community pharmacies are moving to a more clinically focused role, with an increased emphasis on being the first point of call for common ailments and long‐term conditions. To fulfil this expanding role in the area of dermatology, this letter provides an important starting point in identifying some of the challenges that must first be addressed.

## CONFLICT OF INTEREST STATEMENT

The authors declare no conflicts of interest.

## AUTHOR CONTRIBUTIONS

**Zakia Shariff**: Data curation (lead); formal analysis (lead); investigation (lead); methodology (lead); project administration (lead); resources (lead); visualization (lead); writing—original draft (lead); writing—review and editing (lead). **Jane Harvey**: Writing—review and editing (equal). **Paul Leighton**: Writing—review and editing (equal). **Matthew Boyd**: Writing—review and editing (equal). **Matthew Ridd**: Writing—review and editing (equal). **Miriam Santer**: Writing—review and editing (equal). **Rod Tucker**: Writing—review and editing (equal). **Ian Maidment**: Conceptualization (lead); methodology (lead); project administration (equal); supervision (lead); writing—review and editing (equal).

## FUNDING INFORMATION

This article received no specific grant from any funding agency in the public, commercial, or not‐for‐profit sectors.

## ETHICS STATEMENT

This study received ethical approval from the Aston University Research Ethics Committee.

## Data Availability

The data underlying this article will be shared on reasonable request to the corresponding author.

## References

[1] Schofield J , Grindlay D , Williams H . Skin conditions in the UK: a health care needs assessment. https://www.nottingham.ac.uk/research/groups/cebd/documents/hcnaskinconditionsuk2009.pdf. Accessed 19 Aug 2022.

[2] The King’s Fund . How can dermatology services meet current and future patient needs while ensuring that quality of care is not compromised and that access is equitable across the UK? https://kingsfund.blogs.com/health_management/2015/05/how‐can‐dermatology‐services‐meet‐current‐and‐future‐patient‐needs‐while‐ensuring‐that‐quality‐of‐ca.html. Accessed 2 Feb 2022.

[3] Maidment I , Young E , MacPhee M , Booth A , Zaman H , Breen J , et al. Rapid realist review of the role of community pharmacy in the public health response to COVID‐19. BMJ Open. 2021;11(6):e050043. 10.1136/bmjopen-2021-050043 PMC8210681 34135054

[4] Tucker, R . An exploratory study of UK community pharmacists’ perceptions of the skin conditions they encounter: prevalence, reasons for referral and over‐the‐counter dermatological wish list. Self‐care. 2013; 4 (1), 3, 17.

[5] Tucker R . Community pharmacists' perceptions of the skin conditions they encounter and how they view their role in dermatological care. Int J Pharm Pract. 2012;20(5):344–346. 10.1111/j.2042-7174.2012.00212.x 22953774

[6] Leung L , Soeyonggo T . Approach to patient with a generalized rash. J Fam Med Prim Care. 2013;2(4):311–314. 10.4103/2249-4863.123775 PMC4649881 26664833

[7] Ahn CS , Culp L , Huang WW , Davis SA , Feldman SR . Adherence in dermatology. J Dermatol Treat. 2017;28(2):94–103. 10.1080/09546634.2016.1181256 27180785

[8] Tucker R , Stewart D . An exploratory study of the views of community pharmacy staff on the management of patients with undiagnosed skin problems. Int J Pharm Pract. 2015;23(6):390–398. 10.1111/ijpp.12179 25689029

[9] Harvey J , Shariff Z , Anderson C , Boyd MJ , Ridd MJ , Santer M , et al. How can community pharmacists be supported to manage skin conditions? A multistage stakeholder research prioritisation exercise. BMJ Open. 2024;14(1):e071863. 10.1136/bmjopen-2023-071863 PMC10773317 38167282

